# Effectiveness of Saroglitazar in MASLD Patients: A Prospective, Real‐World Assessment of Liver and Metabolic Health

**DOI:** 10.1002/edm2.70144

**Published:** 2025-12-26

**Authors:** Mukulesh Gupta, Harshita Lachhwani, Kumar Praful Chandra, Rajiv Awasthi, Dinesh Kumar

**Affiliations:** ^1^ Udyan Health Care Pvt Ltd Lucknow India; ^2^ Harsha Clinic and Diabetes Centre Lucknow India; ^3^ Chandra Diabetes and Heart Clinic Lucknow India; ^4^ Prarthana Clinic & Diabetes Care Center Lucknow India

**Keywords:** AST, dyslipidemia, MASLD, Saroglitazar

## Abstract

**Background:**

Metabolic Dysfunction‐Associated Steatotic Liver Disease (MASLD) is a significant health concern and is commonly associated with conditions such as dyslipidemia, insulin resistance, and increased risk of cardiovascular disease. Managing MASLD requires addressing both liver and metabolic dysfunction. Saroglitazar, a dual PPARα/γ agonist, has shown potential in addressing liver steatosis, fibrosis, and dyslipidemia.

**Methodology:**

This prospective, single‐arm, multicentric study with 50 MASLD patients with a mean age of 51.84 ± 10.66 years included 33 males. Patients received Saroglitazar magnesium 4 mg in addition to the standard of care for 6 months. The primary objective was to assess changes in liver stiffness measurement (LSM) and controlled attenuation parameter (CAP), and secondary objectives included evaluating changes in metabolic parameters such as fasting blood glucose (FBG), postprandial blood glucose (PPBG), HbA1c, triglyceride levels, and liver enzymes (ALT, alanine aminotransferase; AST, aspartate aminotransferase) at baseline and the end of the study.

**Results:**

A statistically significant improvement in hepatic parameters, including LSM and CAP scores, was observed. Concurrently, at the end of the study duration, 16% of patients showed improvement from liver fibrosis stages of F3/F4 to F0/F1/F2 (*p* < 0.0001), and 76% of patients with severe steatosis (S3) decreased to 38% (*p* < 0.0001). The key metabolic parameters also showed statistically significant reduction in FBG from 140.25 ± 51.5 mg/dL to 117.66 ± 18.17 mg/dL (*p* = 0.004), HbA1c from 7.46% ± 1.44% to 6.83% ± 1.08% (*p* = 0.0004) and triglyceride levels from 238.67 ± 168.35 mg/dL to 167.9 ± 113.89 mg/dL (*p* = 0.0001). However, during the study, anthropometric parameters remained stable, with a minor increase in BMI (28.91 ± 3.5 to 29.12 ± 3.67 Kg/m^2^).

**Conclusion:**

Despite a slight increase in BMI, Saroglitazar significantly improved transient elastography parameters and hepatic parameters in MASLD patients, suggesting that this drug alone effectively manages MASLD‐related metabolic and hepatic dysfunctions.

## Graphical abstract

Saroglitazar 4 mg daily for 6 months significantly improved liver stiffness, steatosis, and metabolic parameters in patients with MASLD in this prospective, real‐world study. The drug demonstrated meaningful regression of fibrosis and steatosis with stable BMI, highlighting its dual hepatic and metabolic benefits in MASLD management.

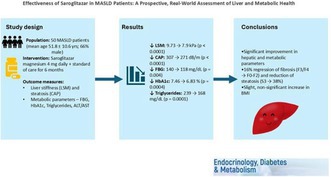

## 1 Introduction

The term Non‐alcoholic Fatty Liver Disease (NAFLD) was first introduced in the 1980s to describe liver fat accumulation not related to excessive alcohol consumption and has since emerged as a significant health concern closely linked to obesity and metabolic syndrome [1]. Over time, as the link between metabolic syndrome and liver disease became clearer, the term Metabolic Dysfunction‐Associated Steatotic Liver Disease (MASLD) was introduced in 2023, replacing NAFLD to better reflect the condition's metabolic origins [2, 3]. MASLD affects approximately 30% of the global adult population, with its prevalence rising from 22% to 37% between 1991 and 2019 [4]. While cardiovascular disease is the leading cause of mortality in MASLD, liver‐related events, especially in patients with advanced fibrosis (F3/F4), pose significant risks [5]. MASLD has also become a leading cause of liver transplantation and hepatocellular carcinoma, with its burden expected to increase globally [6].

MASLD is highly prevalent, with population‐based studies showing that approximately 27% of individuals in Hong Kong have the condition, and 4% have advanced liver fibrosis [7]. The disease often remains asymptomatic until cirrhosis‐related complications arise, making early detection and referral essential [8]. Early identification allows timely intervention to prevent severe outcomes like liver failure or hepatocellular carcinoma [9]. Among high‐risk individuals with T2DM, most have steatotic liver disease, and many also have advanced liver fibrosis. T2DM is recognised as an independent risk factor for MASLD [10, 11].

Calorie restriction and increased physical activity are highly encouraged for MASLD management, with studies showing resolution in up to 90% of patients and improvement in liver fibrosis in up to 45% [12, 13]. However, medications like pioglitazone, glucagon‐like peptide‐1 receptor agonists (GLP‐1), and sodium‐glucose co‐transporter‐2 (SGLT2) inhibitors have shown potential. GLP‐1 RAs, such as liraglutide and semaglutide, have shown effectiveness in resolving MASLD and promoting weight loss, while SGLT2 inhibitors offer cardiometabolic benefits and reduce hepatic fat, though histological data are limited [14, 15, 16]. Pioglitazone, a PPAR‐γ (peroxisome proliferator‐activated receptor‐γ) agonist, improves insulin sensitivity and reduces hepatic triglyceride content but has risks like edema and weight gain [16].

Saroglitazar is a novel dual PPAR α/γ agonist with a strong PPAR‐α effect and moderate PPAR‐γ effect [17]. Approved in India since 2013 for treating diabetic dyslipidemia and hypertriglyceridemia in T2DM patients not controlled by statins, it received additional approvals in 2020 for use in type 2 diabetes and non‐cirrhotic NASH [18, 19]. Saroglitazar is the first and only dual PPAR α/γ agonist approved for clinical use globally, demonstrating beneficial effects on lipid profiles and glycemic control with minimal safety concerns, including weight gain or edema [20].

This study aims to evaluate the effectiveness of Saroglitazar in improving liver and metabolic health in patients with MASLD. The study focuses on the impact of Saroglitazar on liver stiffness, steatosis, and key metabolic parameters over a six‐month period. By examining changes in both transient elastography parameters and hepatic parameters, this study provides insights into the therapeutic potential of Saroglitazar as a treatment for MASLD. This study was previously presented as an abstract at the IDF 2025 conference on April 7, 2025.

## 2 Methodology

### 2.1 Study Design and Participants

This was a multicentric, prospective, open‐label, single‐arm, real‐world clinical study conducted across four centers in India between October 2023 and March 2024. The study included 50 patients aged 18 to 70 years diagnosed with MASLD, as defined by the presence of hepatic steatosis confirmed through controlled attenuation parameter (CAP) and liver stiffness measurement (LSM) using vibration‐controlled transient elastography (Fibroscan). Eligible participants had a Body Mass Index (BMI) ≥ 23 kg/m^2^ and at least one metabolic syndrome parameter (hypertension, diabetes, obesity, dyslipidemia). In addition, all patients had disturbed liver function tests, specifically elevated SGOT and/or SGPT levels, indicating underlying hepatic inflammation. Key exclusion criteria included individuals with established cirrhosis, Type 1 diabetes mellitus, known alcohol consumption beyond the limits defined by the American Association for the Study of Liver Diseases (AASLD), and patients receiving medications known to affect liver health, specifically pioglitazone, vitamin E, and ursodeoxycholic acid (UDCA). Pregnant and lactating women were also excluded.

### 2.2 Intervention

Participants received Saroglitazar magnesium 4 mg once daily, administered orally, in addition to the standard of care for treating MASLD. The standard of care included routine lifestyle modification advice and any antidiabetic drug needed to keep the blood glucose under control, but no additional pharmacological treatments for liver steatosis were permitted during the study period. The treatment duration was 6 months, and patients were evaluated at baseline and at 6 months.

### 2.3 Outcomes

Primary outcome: Change in LSM and CAP from baseline to the end of the study, as assessed by transient elastography.

Secondary outcome: Change in fasting blood glucose (FBG), glycated haemoglobin (HbA1c), triglyceride levels, and liver enzymes (Aspartate aminotransferase; AST, Alanine aminotransferase; ALT) at baseline and the end of the study.

### 2.4 Sample Size Calculation

No formal sample size calculation was performed for this study, as it was designed as a time‐bound, real‐world assessment of Saroglitazar in patients with MASLD. The recruitment window was predefined (October 2023 to March 2024), and during this period, all patients meeting the inclusion and exclusion criteria at the participating centers were enrolled. This approach reflects the pragmatic, exploratory intent of the study, focusing on evaluating treatment effectiveness under routine clinical practice.

### 2.5 Data Collection and Statistical Analysis

Data were collected from 50 MASLD patients over 6 months, with assessments conducted at baseline and the end of the study. Demographic information, LSM, CAP, and metabolic markers such as FBG, PPBG, HbA1C, and lipid profiles were recorded. Statistical analysis was performed using paired *t*‐tests to compare mean changes in these parameters between visits, and chi‐square tests were used to analyse the change in LSM, CAP, and triglyceride levels. Significance was defined as *p* < 0.05, with results showing statistically significant improvements in most parameters.

## 3 Results

The baseline characteristics of the study population, including age, height, weight, and gender distribution, are summarised in Table 1.

**TABLE 1:** Patient demographic data.

Parameters	Mean ± SD
Age (years)	51.84 ± 10.66
Height (cm)	161.41 ± 10.82
Weight (kgs)	76.26 ± 12.91

Gender	Frequency *n* (%)
Female	17 (34)
Male	33 (66)

### 3.1 Changes in Liver Parameters

**FIGURE 1:**
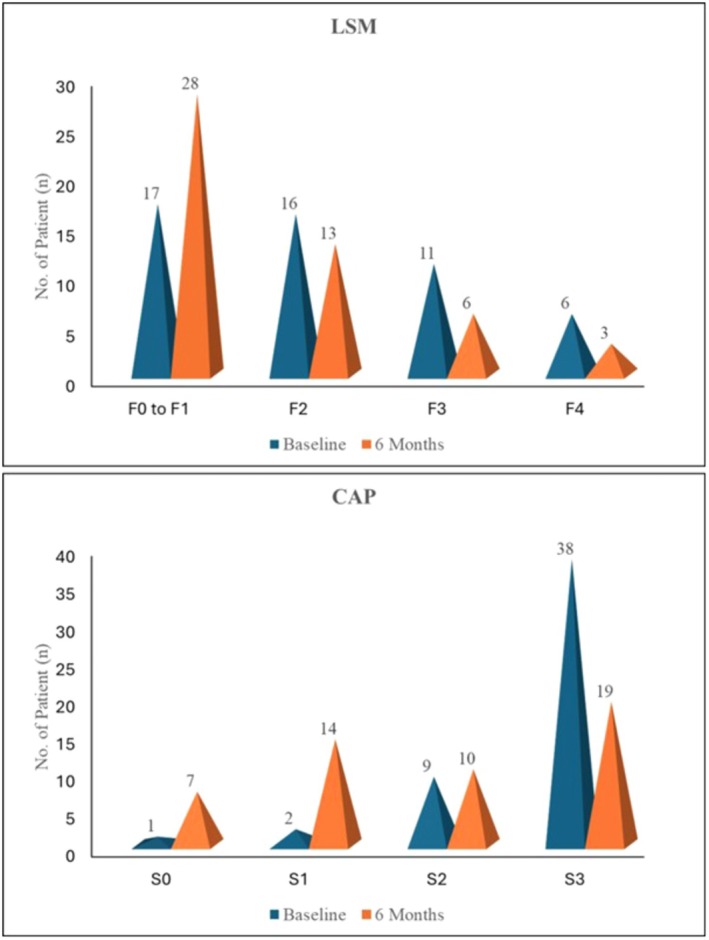
(A) Distribution of patients across different liver stiffness measurement (LSM) stages at baseline (Visit‐1) and 6 Months (Visit‐5). (B) Distribution of patients across steatosis grades (CAP scores) at baseline (Visit‐1) and 6 Months (Visit‐5).

At baseline, advanced liver fibrosis (F3/F4) was observed in 17 patients, indicating significant fibrotic involvement in the study cohort. Following 6 months of Saroglitazar magnesium 4 mg treatment, this number declined to 9 patients (*p* = 0.02) (Figure 1A), reflecting a notable regression in fibrosis stages (Table 2). These improvements were accompanied by a significant decrease in mean LSM scores (*p* < 0.0001). Similarly, severe hepatic steatosis (S3) was present in 38 patients at baseline. By the end of the study period, this number had reduced to 19 patients (*p* < 0.0001), demonstrating a marked reduction in severe steatosis (Figure 1B). A corresponding decrease in mean controlled attenuation parameter (CAP) scores was also observed, which was statistically significant (*p* < 0.0001).

**TABLE 2:** Change in LSM and CAP score at baseline and end of the study.

Parameters	Baseline (mean ± SD)	6 Months (mean ± SD)	Paired *t*‐test
LSM score (kPa)	9.73 ± 4.75	7.9 ± 3.27	*p* < 0.0001
CAP score (dB/m)	307.3 ± 31.24	271.46 ± 33.26	*p* < 0.0001

### 3.2 Change in Clinical Parameters

Significant improvements were observed in several clinical parameters, including reductions in FBG, PPBG, HbA1c, ALT, and triglyceride levels (*p* < 0.01). However, no significant changes were noted in BMI, platelet count, AST, HDL, or FIB‐4 score (Table 3). The proportion of patients with hypertriglyceridemia (≥ 200 mg/dL) decreased significantly from 52% to 20% (*p* = 0.006).

**TABLE 3:** Change in clinical parameters at baseline and end of study.

Parameters	Baseline (mean ± SD)	End of study (mean ± SD)	Paired *t*‐test
BMI (kg/m^2^)	28.91 ± 3.5	29.12 ± 3.67	*p* = 0.23
FBG (mg/dL)	140.25 ± 51.5	117.66 ± 18.17	*p* = 0.004*
PPBG (mg/dL)	200.44 ± 68.19	161.32 ± 50.51	*p* = 0.0004*
HbA1c (%)	7.46 ± 1.44	6.83 ± 1.08	*p* = 0.0004*
Platelets (×10^9^/L)	159.89 ± 76.15	164.5 ± 77.92	*p* = 0.39
ALT (U/L)	47.4 ± 36.83	34.41 ± 23.19	*p* = 0.002*
AST (U/L)	38 ± 24.23	31.97 ± 12.27	*p* = 0.06
Triglyceride (mg/dL)	238.67 ± 168.35	167.9 ± 113.89	*p* = 0.0001*
HDL (mg/dL)	44.77 ± 10.53	47.42 ± 12.19	*p* = 0.12
FIB‐4 score	1.7 ± 0.93	1.65 ± 0.69	*p* = 0.44
TyG index	5.1 ± 0.4	4.87 ± 0.29	*p* < 0.0001

Abbreviations: ALT, alanine aminotransferase; AST, aspartate aminotransferase; BMI, Body Mass Index; FBG, fasting blood glucose; FIB‐4, fibrosis‐4 index; HbA1c, glycated haemoglobin; HDL, high density lipids; PPBG, postprandial blood glucose; TyG, triglyceride‐glucose index. *Note*: * Indicates statistically significant difference between baseline and end‐of‐study values (p < 0.05).

## 4 Discussion

The present study shows that Saroglitazar magnesium significantly improves both liver health and metabolic parameters in patients with MASLD over 6 months. The present study observed a statistically significant reduction in mean CAP and LSM scores of 36 dB/m and 1.83 kPa, respectively. Similarly, in a study done by Goyal et al. [21], treatment with Saroglitazar led to a 0.9 kPa reduction in LSM (from 8.4 to 7.5 kPa) and a 79 dB/m reduction in CAP (from 335 to 256 dB/m) after 24 weeks of treatment. The coexistence of MASLD and T2DM is common and known to increase microvascular complications of diabetes, such as chronic kidney disease and retinopathy, often leading to mortality [22] Further, the results of the present study demonstrate a significant improvement in glycemic control among patients treated with Saroglitazar. The observed reduction in HbA1c levels from 7.46% at baseline to 6.83% at the end of the study showed a mean HbA1c reduction of 0.63% with Saroglitazar treatment [23].

The improvement in lipid profile is a crucial aspect of managing MASLD, as dyslipidemia is a major contributor to both liver disease progression and cardiovascular risk in these patients. Elevated triglycerides and abnormal cholesterol levels are frequently seen in MASLD, and they exacerbate hepatic fat accumulation, leading to further inflammation, fibrosis, and the eventual progression to more severe liver conditions, such as cirrhosis [24, 25]. In the Goyal et al. [21] study, Saroglitazar treatment over 24 weeks resulted in a 48.4% reduction in triglyceride levels, decreasing from 326.4 mg/dL to 168.3 mg/dL. Similarly, Jani et al. (2014) reported a 46.7% reduction in triglycerides, highlighting the consistent lipid‐lowering effect of Saroglitazar across different patient populations [23]. A case report by Kulkarni et al. (2024) showed a 48% reduction in triglycerides after 24 weeks of Saroglitazar treatment in a MASLD patient. The CAP score also showed a marked decrease from 330 dB/m to 241 dB/m, indicating a significant reduction of hepatic steatosis [26].

The improvements observed in liver and metabolic parameters in MASLD patients treated with Saroglitazar can be attributed to its dual action as a PPAR‐α/γ agonist, which targets both lipid metabolism and insulin sensitivity [27]. Activation of PPAR‐α promotes fatty acid oxidation, reducing hepatic triglyceride synthesis and accumulation, leading to decreased hepatic steatosis and inflammation. This mechanism also reduces oxidative stress and inflammation, key factors in preventing the progression of steatosis to fibrosis [28]. Simultaneously, PPAR‐γ activation enhances insulin sensitivity by upregulating insulin‐responsive genes, improving glycemic control, and reducing insulin resistance, which is central to MASLD pathology. This dual activation also decreases lipotoxic species such as ceramides and diglycerides, further mitigating liver inflammation and fibrosis [29, 30]. In addition, a statistically significant reduction was observed in the triglyceride‐glucose (TyG) index, from 5.1 ± 0.4 to 4.87 ± 0.29 (*p* < 0.0001). As an emerging surrogate marker of insulin resistance, the TyG index has been shown to predict metabolic dysfunction and hepatic steatosis; thus, its reduction further supports the metabolic benefits of Saroglitazar [31, 32]. These improvements demonstrate the effectiveness of Saroglitazar in addressing both hepatic and metabolic dysfunctions in MASLD.

An essential aspect of this study is that no specific lifestyle changes or exercise interventions were prescribed beyond routine advice. Despite the slight increase in BMI, which was not statistically significant, the effectiveness of Saroglitazar was evident through substantial improvements in both clinical and hepatic parameters. This highlights the effectiveness of the drug in improving liver stiffness, steatosis, and metabolic markers, independent of weight loss or targeted lifestyle modifications. However, the study has several limitations, including the small sample size, lack of a control group, and a relatively short follow‐up period.

## 5 Conclusion

In this study, Saroglitazar demonstrated significant effectiveness in improving both liver and metabolic health in patients with MASLD. The shift in patients from advanced fibrosis and severe steatosis to milder stages further highlights its therapeutic potential. Given its dual action on PPAR α/γ, Saroglitazar presents a promising option for managing MASLD. It addresses both hepatic and metabolic dysfunctions without significant safety concerns, making it a valuable addition to current treatment strategies.

## Author Contributions

**Dinesh Kumar:** conceptualization, investigation, funding acquisition, writing – original draft, methodology, validation, visualization, writing – review and editing, project administration, supervision. **Mukulesh Gupta:** data curation, investigation, resources, methodology, writing – review and editing. **Harshita Lachhwani:** formal analysis, data curation, literature review, writing – review and editing. **Kumar Praful Chandra:** software, data management, validation, visualization, writing – review and editing. **Rajiv Awasthi:** investigation, resources, critical revision of manuscript, writing – review and editing.

## Funding

The authors have nothing to report.

## Ethics Statement

All patients provided informed consent before enrolment, and the study was conducted in compliance with the Declaration of Helsinki. The study protocol was approved by the institutional ethics committees at each participating centre (Approval ID: HCDC/03/2023). The study was registered prospectively in the Clinical Trials Registry of India (CTRI/2023/10/059025).

## Consent

All patients provided informed consent before enrolment, and the study was conducted in compliance with the Declaration of Helsinki.

## Conflicts of Interest

The authors declare no conflicts of interest.

## Data Availability

The data that support the findings of this study are available from the corresponding author upon reasonable request.
